# Potential Impact of Doxycycline Post-exposure Prophylaxis Prescribing Strategies on Incidence of Bacterial Sexually Transmitted Infections

**DOI:** 10.1093/cid/ciad488

**Published:** 2023-08-18

**Authors:** Michael W Traeger, Kenneth H Mayer, Douglas S Krakower, Sy Gitin, Samuel M Jenness, Julia L Marcus

**Affiliations:** Department of Population Medicine, Harvard Medical School and Harvard Pilgrim Health Care Institute, Boston, Massachusetts, USA; The Fenway Institute, Fenway Health, Boston, Massachusetts, USA; Disease Elimination Program, Burnet Institute, Melbourne, Victoria, Australia; The Fenway Institute, Fenway Health, Boston, Massachusetts, USA; Division of Infectious Diseases, Department of Medicine, Beth Israel Deaconess Medical Center, Boston, Massachusetts, USA; Department of Population Medicine, Harvard Medical School and Harvard Pilgrim Health Care Institute, Boston, Massachusetts, USA; The Fenway Institute, Fenway Health, Boston, Massachusetts, USA; Division of Infectious Diseases, Department of Medicine, Beth Israel Deaconess Medical Center, Boston, Massachusetts, USA; The Fenway Institute, Fenway Health, Boston, Massachusetts, USA; Department of Epidemiology, Emory University, Atlanta, Georgia, USA; Department of Population Medicine, Harvard Medical School and Harvard Pilgrim Health Care Institute, Boston, Massachusetts, USA; The Fenway Institute, Fenway Health, Boston, Massachusetts, USA

**Keywords:** sexually transmitted infections, doxycycline post-exposure prophylaxis, doxypep, STI prevention, HIV

## Abstract

**Background:**

Doxycycline post-exposure prophylaxis (doxyPEP) reduces bacterial sexually transmitted infection (STI) incidence in people with HIV (PWH) or using HIV pre-exposure prophylaxis (PrEP). Given concerns about widespread antibiotic use, we identified doxyPEP prescribing strategies to minimize use while maximizing impact on STIs.

**Methods:**

We used electronic health records of gay and bisexual men (GBM), transgender women, and nonbinary people assigned male sex at birth with ≥2 STI tests (chlamydia, gonorrhea, syphilis) at an LGBTQ-focused health center during 2015–2020. We defined 10 hypothetical doxyPEP prescribing strategies based on PrEP use, HIV status, or STI history. We estimated doxyPEP use and STI diagnoses averted in counterfactual scenarios in which people meeting prescribing criteria received doxyPEP, assuming STI rates during use would have been reduced by clinical trial efficacy estimates.

**Results:**

Among 10 546 individuals (94% GBM), rate of any STI was 35.9/100 person-years. Prescribing doxyPEP to all individuals would have averted 71% of STI diagnoses (number needed to treat for one year to avert one STI diagnosis [NNT] = 3.9); prescribing to PrEP users/PWH (52%/12% of individuals) would have averted 60% of STI diagnoses (NNT = 2.9). Prescribing doxyPEP for 12 months after STI diagnosis would have reduced the proportion using doxyPEP to 38% and averted 39% of STI diagnoses (NNT = 2.4). Prescribing after concurrent or repeated STIs maximized efficiency (lowest NNTs) but prevented fewer STIs.

**Conclusions:**

Prescribing doxyPEP to individuals with STIs, particularly concurrent or repeated STIs, could avert a substantial proportion of all STI diagnoses. The most efficient prescribing strategies are based on STI history rather than HIV status or PrEP use.

## Teaser

Using electronic health records, we assessed hypothetical doxycycline post-exposure prophylaxis prescribing strategies. Doxycycline could avert a substantial proportion of sexually transmitted infection (STI) diagnoses, with the most efficient strategies based on STI history, not HIV status or pre-exposure prophylaxis use.

Sexually transmitted infections (STIs) are a major cause of morbidity globally, with approximately 370 million diagnoses of bacterial STIs occurring each year [1]. Bacterial STIs continue to increase in the United States, most notably among gay and bisexual men (GBM), transgender people, and young heterosexual people [2]. Drivers of increasing STI incidence include behavioral, structural, and social factors. Reductions in condom use [3] and changes in sexual networks among GBM [4] have occurred alongside greater awareness that human immunodeficiency virus (HIV) treatment decreases forward transmission (known as treatment as prevention) [5] and increased use of HIV pre-exposure prophylaxis (PrEP), which is highly effective at preventing HIV acquisition, irrespective of condom use [6].

Beyond screening and treatment, innovative approaches to control STIs are needed. Clinical trials have evaluated doxycycline, a moderate-spectrum tetracycline antibiotic that is rapidly absorbed after oral administration, as post-exposure prophylaxis for bacterial STIs among people assigned male sex at birth. A randomized, open-label, clinical trial of 232 GBM in France who were using on-demand HIV PrEP found that taking 200 mg of doxycycline within 24 hours after condomless sex reduced chlamydia incidence by 70% and syphilis incidence by 73%, with no effect on gonorrhea [7]. A more recent trial in the United States explored 200 mg doxycycline within 72 hours after condomless sex among people with HIV (PWH) or those taking HIV PrEP; the incidence of chlamydia was reduced by 74–88%, syphilis by 77–87%, and gonorrhea by 55–57% [8]. In both of these studies, doxycycline was well tolerated and self-reported adherence was high.

The development of guidance for doxycycline post-exposure prophylaxis (doxyPEP) prescribing is complicated by concerns about potential harms of widespread antibiotic use. DoxyPEP has the potential to promote antimicrobial resistance and long-term side effects associated with antibiotic consumption, such as alteration in the individual or community microbiome that could facilitate colonization with pathogens such as antibiotic-resistant *Staphylococcus aureus* or *Clostridioides difficile* [9]. DoxyPEP prescribing guidelines will therefore need to define strategies that minimize overall antibiotic prescribing while maximizing impact on STI incidence. To inform implementation of doxyPEP, we evaluated the potential impact and efficiency of hypothetical doxyPEP prescribing strategies, including prescribing to populations defined by HIV PrEP use, diagnosed HIV status, or current or prior STI diagnosis.

## METHODS

### Study Setting and Population

We extracted data from electronic health records (EHRs) at Fenway Health, a large federally qualified community health center in Boston, Massachusetts, that specializes in care for sexual and gender minorities [10]. The study population included GBM, transgender women, and nonbinary people who were assigned male sex at birth, aged 18 years or older, and tested at least twice for STIs (chlamydia, gonorrhea, or syphilis) from 1 January 2015 through 31 December 2020.

### Data Extraction

The EHR variables included demographics (age, sex assigned at birth, gender identity, sexual orientation, race, ethnicity), encounter dates, STI test dates and results, HIV test dates and results, HIV PrEP prescription dates, and HIV diagnosis dates.

### Incidence Analyses

We used repeat-testing methods [11] to calculate diagnosis rates for chlamydia, gonorrhea, and syphilis individually, as well as for any STI. Individuals contributed person-time from their first STI test during the study period and were censored at their last STI test or 31 December 2020, whichever occurred first. The diagnosis rate of any STI was calculated for 3 groups—PWH, people prescribed PrEP, and people without HIV and not prescribed PrEP—and was defined as the number of new diagnoses divided by the total number of person-years accrued in each group and expressed as rates per 100 person-years. People were classified as diagnosed with HIV from the date of first recorded HIV diagnosis date onwards and as PrEP users from the date of first recorded PrEP prescription. New cases of gonorrhea and chlamydia were defined by positive nucleic acid amplification tests or, for gonorrhea, a positive culture. New cases of syphilis were defined using a previously validated algorithm based on rapid plasma reagin tests for detecting new cases of infectious (primary, secondary, or early latent) syphilis [12, 13] (Supplementary Appendix 1). STI diagnoses on the date of study entry were not included in the numerator for STI diagnosis rates. For the outcome of any STI, concurrent positive results for the same pathogen at multiple anatomical sites (eg, urethral chlamydia and rectal chlamydia) were considered a single infection, while concurrent infections of different pathogens were considered multiple infections regardless of anatomic site (eg, rectal gonorrhea and rectal chlamydia). For each subpopulation, we also calculated the median time between test events (visits where any STI test was performed), as well as the mean number of test events per person in each year and across the study period.

### DoxyPEP Prescribing Strategies

We evaluated the potential impact and efficiency of 10 hypothetical doxyPEP prescribing strategies. We first evaluated 3 strategies in which doxyPEP would be prescribed indefinitely to the following groups defined by HIV status and use of PrEP:

1. All individuals (from their first STI test)
2. All people diagnosed with HIV (from date of HIV diagnosis or from cohort entry if diagnosis was prior to 2015) and all PrEP users (from first PrEP prescription)
3. All PrEP users only (from first PrEP prescription)

Scenarios prescribing doxyPEP to just PrEP users were considered as PrEP users had the highest STI incidence, followed by PWH.

We also evaluated 7 hypothetical prescribing strategies in which doxyPEP would be prescribed for 12 months to individuals meeting criteria based on STI results at the current testing visit and STI history as documented in the EHR (STI-based strategies; in descending order by frequency of people meeting the prescribing criteria):

4. Diagnosis of any STI at current visit
5. Diagnosis of a rectal STI (chlamydia or gonorrhea) at current visit
6. Diagnosis of gonorrhea at current visit
7. Diagnosis of any STI at current visit and diagnosis of any STI in past 12 months
8. Diagnosis of any STI at current visit and diagnosis of any STI in past 6 months
9. Diagnosis of syphilis at current visit
10. Concurrent diagnoses of at least 2 STIs at current visit

In these strategies, individuals could be prescribed doxyPEP multiple times if they met prescribing criteria at a subsequent visit at least 12 months after previously meeting the criteria.

Finally, we evaluated strategies 4–10 restricted to patients who met the STI-based criteria and were also (a) a recent PrEP user (<6 months since last prescription) or a person diagnosed with HIV or (b) a recent PrEP user.

### Estimating DoxyPEP Use, Impact, and Efficiency

We evaluated counterfactual scenarios in which individuals who met the criteria for each doxyPEP prescribing strategy were assumed to have been prescribed (and to have used) doxyPEP within 72 hours after each episode of condomless sex. In these counterfactual scenarios, periods of person-time following a hypothetical doxyPEP prescription were classified as “doxyPEP periods,” which ended after 12 months for strategies 4–10 or when individuals were censored, whichever came first.

To estimate the impact of doxyPEP on STI diagnosis rates under each prescribing strategy, we estimated the proportion of diagnoses that would have been prevented by doxyPEP if STI diagnoses during doxyPEP periods were reduced by trial efficacy estimates, including specific reductions for each STI and subgroup (ie, PrEP users and PWH) [8] (Supplementary Appendix 2). Diagnoses were classified as being in doxyPEP periods if the midpoint between diagnosis date and previous negative test was within 12 months of a hypothetical doxyPEP prescription. We estimated the reduction in STI diagnoses, rather than STI transmissions, because we relied on tests and results recorded in EHR data, and because testing rates varied across subgroups. Relative reductions in STI diagnoses across scenarios therefore incorporate the observed testing and diagnosis rates within each subgroup during the study period. We assessed the efficiency of each prescribing strategy by estimating the number needed to treat with doxyPEP for one year (NNT) to avert one STI diagnosis.

Analyses were conducted in STATA version 17.0 (StataCorp, College Station, Texas).

### Ethical Approval

This study was approved by the Institutional Review Board at Fenway Community Health.

## RESULTS

### Participant Characteristics

A total of 10 546 GBM, transgender women, and nonbinary individuals assigned male sex at birth were tested at least twice for chlamydia, gonorrhea, or syphilis during the study period. The median age at first test was 32 years (interquartile range [IQR]: 26–45 y). The majority (86.3%) identified as gay men; 7.3% identified as bisexual men, 4.3% as transwomen, and 2.1% as nonbinary (Table 1). Most (70.8%) identified as White, 6.5% as Black or African-American, 6.2% as multiracial, and 5.9% as Asian or Pacific Islander; 14.6% identified as Hispanic. By the end of the study period, 11.9% of individuals had an HIV diagnosis. Approximately half (52.0%) had a PrEP prescription during the study period.

**Table 1.** Characteristics of Individuals Tested for STIs at Least Twice: Fenway Health, 2015–2020

Characteristic	n (%)
Age in years at first STI test	
18–29	4215 (40.0)
30–39	2773 (26.3)
40–49	1669 (15.8)
50+	1889 (17.9)
Gender identity and sexual orientation	
Gay male	9106 (86.3)
Bisexual male	768 (7.3)
Transgender female	451 (4.3)
Nonbinary	221 (2.1)
Race	
White	7468 (70.8)
Black or African-American	684 (6.5)
Multiracial	654 (6.2)
Asian	624 (5.9)
American Indian or Alaska Native	56 (0.5)
Native Hawaiian or Other Pacific Islander	25 (0.2)
Other	196 (1.9)
Not recorded	839 (8.0)
Ethnicity	
Hispanic	1537 (14.6)
Not Hispanic	8133 (77.1)
Not recorded	876 (8.3)
Diagnosed with HIV by final visit	1254 (11.9)
Prescribed PrEP during the study period	5486 (52.0)

Includes N = 10 546 individuals with at least 2 test events for chlamydia, gonorrhea, or syphilis.

Abbreviations: HIV, human immunodeficiency virus; PrEP, pre-exposure prophylaxis; STI, sexually transmitted infection.

### STI Diagnosis Rate During Study Follow-up

Total follow-up among all participants was 28 275 person-years (median: 2.6 y; IQR: 1.1–4.2 y). There were 10 144 STI diagnoses during the study period, with an overall diagnosis rate of 35.9 per 100 person-years for any STI. The diagnosis rate of any STI was 36.2 per 100 person-years among PWH, 40.3 per 100 person-years among people prescribed PrEP, and 10.8 per 100 person-years among people without HIV and with no history of PrEP prescription (Supplementary Appendix 3). In STI-specific analyses, diagnosis rates were 15.9 per 100 person-years for chlamydia, 13.8 per 100 person-years for gonorrhea, and 3.7 per 100 person-years for syphilis. The median number of days between STI test events was 126 (IQR: 68–217) for PWH, 93 (IQR: 63–135) for people prescribed PrEP, and 173 (IQR: 80–338) for people without HIV and no history of PrEP prescription (see Supplementary Appendix 4 for mean annual testing rates).

### Estimated DoxyPEP Coverage and STI Diagnoses Averted Under DoxyPEP Prescribing Strategies

**Figure 1.**
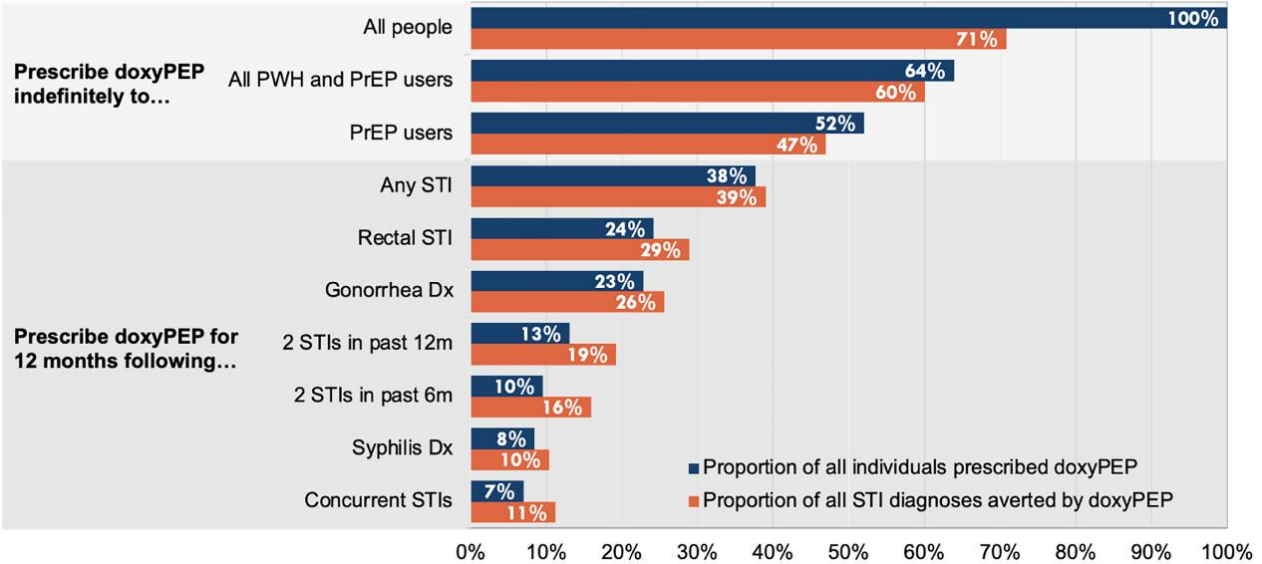
Proportion of individuals prescribed doxyPEP and proportion of STI diagnoses averted in counterfactual scenarios in which each doxyPEP prescribing strategy was assumed to have been implemented. The proportion of STI diagnoses averted is for any STI (chlamydia, gonorrhea, or syphilis). See Supplementary Appendix 6 for the proportion of person-time spent in doxyPEP periods and Supplementary Appendix 7 for the proportion of each STI diagnosis averted in each counterfactual scenario. Abbreviations: doxyPEP, doxycycline post-exposure prophylaxis; Dx, diagnosis; m, months; PrEP, pre-exposure prophylaxis; PWH, people with HIV; STI, sexually transmitted infection.

Figure 1 shows the estimated doxyPEP use and STI diagnoses averted in counterfactual scenarios in which each prescribing strategy was assumed to have been implemented (see Supplementary Appendix 5 for estimated proportion of all person-time spent in doxyPEP periods). In general, as the estimated proportion of people prescribed doxyPEP decreased across strategies, so did the estimated proportion of STI diagnoses averted—ranging from the strategy of prescribing doxyPEP to 100% of people, which would have averted 70.8% of STI diagnoses, to the strategy of prescribing doxyPEP for 12 months following concurrent STI diagnoses, in which 7% of people would have been prescribed doxyPEP and 11.2% of STI diagnoses would have been averted.

Compared with the prescribing strategies in which all people, PrEP users, or both PrEP users and PWH were prescribed doxyPEP (strategies 1–3), strategies that used an STI diagnosis as an indication (strategies 4–10) would have resulted in a higher proportion of STI diagnoses averted relative to the proportion of people prescribed doxyPEP (Figure 1). For example, if doxyPEP were prescribed to all PrEP users and PWH regardless of STI diagnosis, 63.9% of individuals would have been prescribed doxyPEP and 60.0% of STI diagnoses would have been averted. In contrast, prescribing doxyPEP for 12 months following diagnosis of any STI, regardless of PrEP use or HIV status, would have resulted in 37.7% of people being prescribed doxyPEP (41.1% fewer people prescribed doxyPEP and 48.7% less person-time spent on doxyPEP) and 39.4% of STI diagnoses being averted (only 34.3% fewer STIs averted). Similar relative differences were observed when examining the proportion of diagnoses averted for chlamydia and gonorrhea separately. Prescribing doxyPEP following a syphilis diagnosis prevented 19% of subsequent syphilis diagnoses (Supplementary Appendix 7).

### Estimated Efficiency of DoxyPEP Prescribing Strategies

**Figure 2.**
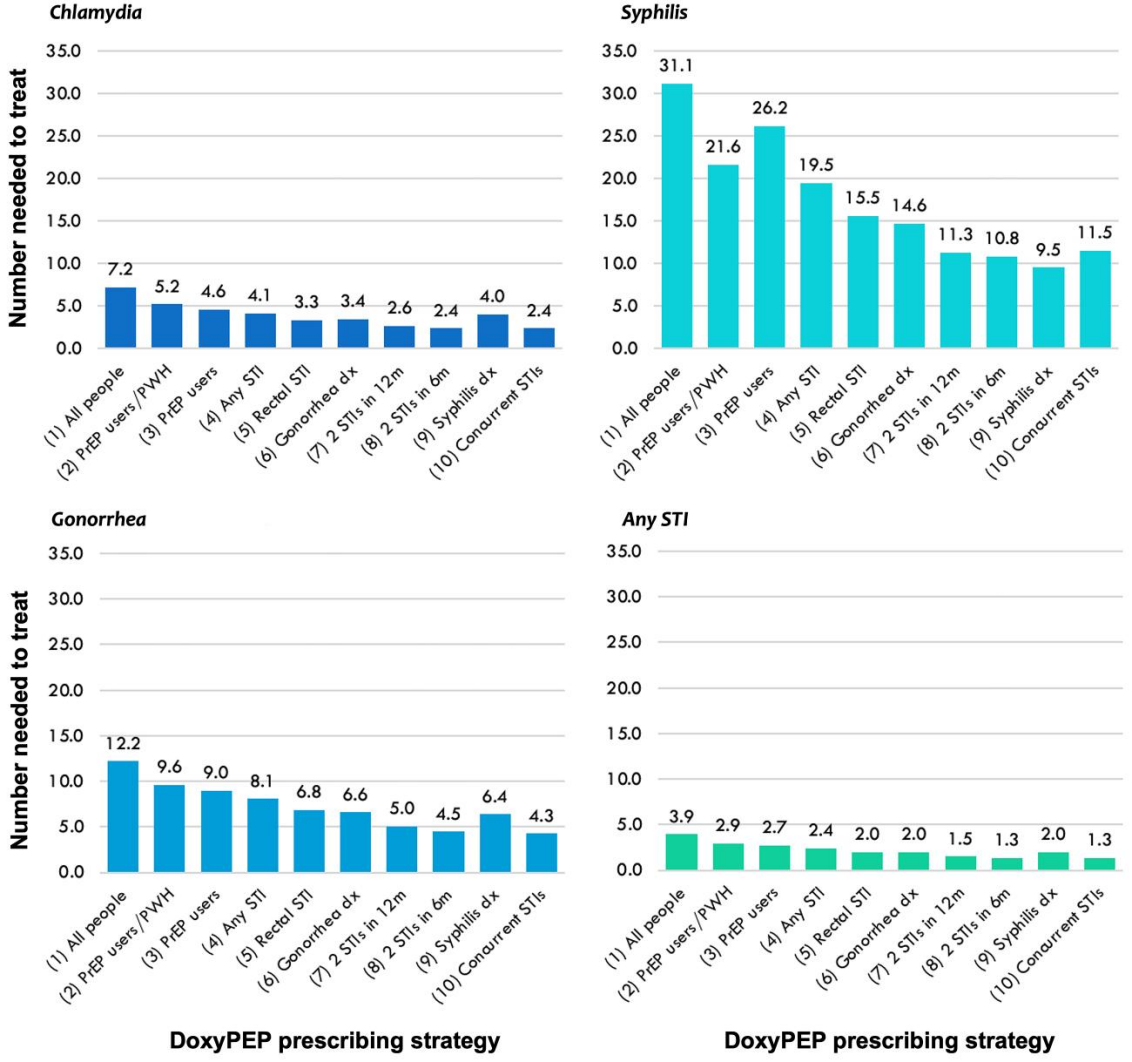
Number of people needed to treat with doxyPEP for one year to avert one diagnosis of chlamydia, syphilis, gonorrhea, and any STI in counterfactual scenarios in which each doxyPEP prescribing strategy was assumed to have been implemented. Strategies 1–3 prescribe doxyPEP indefinitely to respective subgroups. Strategies 4–10 prescribe doxyPEP for 12 months following the respective criteria based on STI diagnosis. The number needed to treat is defined as the total number of person-years of doxyPEP use divided by the total number of STI diagnoses averted in each counterfactual scenario. Abbreviations: doxyPEP, doxycycline post-exposure prophylaxis; dx, diagnosis; m, months; PrEP, pre-exposure prophylaxis; PWH, people with HIV; STI, sexually transmitted infection.

For averting any STI diagnosis, the NNT was 3.9 if doxyPEP were prescribed to all people, 2.9 if prescribed to PrEP users and PWH, and 2.7 if prescribed to PrEP users only (Figure 2). If doxyPEP were prescribed for 12 months following an STI diagnosis, regardless of PrEP use or HIV status, the NNT was lower, ranging from 1.3 to 2.4. Across all prescribing strategies, the NNT ranged from 2.4 to 7.2 for chlamydia, 4.3 to 12.2 for gonorrhea, and 9.5 to 31.1 for syphilis (Figure 2). For preventing chlamydia, gonorrhea, or any STI diagnosis, the lowest NNTs were for strategies in which doxyPEP was prescribed for 12 months following multiple STI diagnoses (ie, 2 STIs within 6 mo, 2 STIs within 12 mo, or concurrent STIs). For preventing syphilis, prescribing doxyPEP for 12 months after a diagnosis of syphilis yielded the lowest NNT (9.5).

### Restricting STI-based Strategies to PrEP Users and Persons With HIV

When restricting strategies that would prescribe doxyPEP for 12 months following one or more STI diagnoses (strategies 4–10) to PrEP users and/or PWH, reductions in the proportion of STI diagnoses averted were generally more pronounced than reductions in the NNT across strategies (Supplementary Appendix 8). For preventing syphilis, restricting doxyPEP following an STI diagnosis to people prescribed PrEP resulted in an increase in the NNT (less efficient) across strategies (Supplementary Appendix 8).

## DISCUSSION

In this cohort of GBM, transgender women, and nonbinary people who were assigned male sex at birth and tested for STIs at an urban community health center, we estimated that prescribing doxyPEP to key subgroups could lead to substantial reductions in STI diagnoses if adherence were similar to that observed in clinical trials. Unsurprisingly, our results suggested that prescribing doxyPEP to more individuals would avert more STI diagnoses; however, given concerns about widespread antibiotic use, we identified prescribing strategies that would minimize doxyPEP prescribing while maximizing the impact on STIs. Compared with prescribing doxyPEP to all PrEP users and PWH, we estimated that prescribing doxyPEP following an STI diagnosis would reduce the total amount of person-time on doxyPEP by almost half, while the proportion of STI diagnoses averted would be reduced by only one-third; this reflects the high rate of STI diagnoses in the 12 months after an STI diagnosis in our cohort. Furthermore, we estimated minimal gains in efficiency when restricting STI-based prescribing strategies to PrEP users or PWH. Our results suggest that clinical guidelines that incorporate 1 or more STI diagnoses in the past 12 months may offer an efficient and straightforward strategy for prescribing doxyPEP, especially for populations experiencing high rates of STIs.

Beyond efficiency, there may be several additional benefits of STI-based prescribing strategies for doxyPEP. Using STI diagnosis as an indication for doxyPEP prescribing—rather than prescribing to PrEP users or PWH—may improve access to doxyPEP for groups that may otherwise have been excluded despite having an increased prospective STI risk (eg, people who do not have HIV, are not using PrEP, and have an STI diagnosis). Moreover, given the racial and ethnic inequities that have been observed in PrEP access and use, doxyPEP prescribing strategies that focus on PrEP users could inadvertently reproduce similar inequities in doxyPEP access and use, whereas STI-based prescribing of doxyPEP may help minimize such inequities.

We estimated that doxyPEP could be a highly efficient strategy for preventing STIs among people at increased risk of STIs, with NNTs ranging from 1.3 to 3.9 to prevent any bacterial STI and up to 31.1 for preventing syphilis. Notably, we observed differences between STIs in the estimated impact and efficiency of doxyPEP prescribing strategies; these differences were driven by heterogeneity in baseline rates of each STI, patterns of repeat diagnosis for each STI, and efficacy of doxyPEP in reducing each STI. Although gains in efficiency (lower NTTs) generally corresponded with reduced impact on STIs, prescribing doxyPEP following a syphilis diagnosis yielded the lowest NNT for averting syphilis diagnoses (9.0) but had a slightly greater estimated impact on syphilis diagnoses than other strategies. Public health efforts aimed specifically at reducing the burden of syphilis should consider prior syphilis diagnosis as an indication for doxyPEP.

There are concerns that antimicrobial resistance in bacterial STIs may increase through selection of resistant strains following broader uptake of doxyPEP [9, 14]. Although there has been no evidence of doxycycline resistance in chlamydia or syphilis [15, 16], gonorrhea is becoming increasingly resistant to ceftriaxone and azithromycin, which are used for first- and second-line treatment of gonorrhea, respectively. Tetracycline resistance in cases of gonorrhea in the United States is estimated to be 25% [17], while ceftriaxone resistance is rare, at 0.2% [18]. Clinical trials of doxyPEP showed an increase in tetracycline resistance in gonorrhea isolates among participants, from 20% at baseline to 40% after 1 year of follow-up [8]. However, an anticipated benefit of doxyPEP is a reduction in the amount of antibiotics prescribed to treat STIs. The use of doxyPEP to prevent gonorrhea in settings where tetracycline resistance remains moderate or low may reduce the number of people who need to be treated, slowing resistance to first-line treatment drugs; such benefits may be more limited in settings with high tetracycline resistance, including in some European countries where resistance in gonorrhea cases is estimated to be 60–70% [19, 20]. Modelling suggests that, while doxyPEP may reduce the burden of gonorrhea in the short term, selection for doxycycline-resistant strains would likely lead to a loss in prophylactic benefit [21]. DoxyPEP use may also select for resistance for other antibiotics, as resistant genes are often linked and transmitted together [22]. Monitoring for antimicrobial resistance in gonorrhea and other infections (eg, *Staphylococcus aureus)* among people using doxyPEP will be important.

Modelling suggests that the impact of doxyPEP on population-level STI incidence will depend not only on appropriate implementation focused on specific subgroups at increased risk of STIs but also on uptake and persistence [23]. Although interest in doxyPEP among GBM is high [24], real-world use is yet to be determined. Monitoring uptake, adherence, and impact during the initial phase of doxyPEP implementation will be critical for refining guidelines and clinical practice. Other potential effects of doxyPEP should be considered in modelling and observational studies, including the impact on consumption of antibiotics, prevention of STI-related sequelae, and effects on users' sexual well-being [25].

### Limitations

There are several limitations to our analysis. First, our estimates were from one health care center that specializes in the care of sexual and gender minorities and may not generalize to all populations at increased risk of STIs. Most individuals in our cohort were White and non-Hispanic, and there were few transgender and nonbinary individuals, reflecting the demographics of the population accessing STI testing at Fenway Health. Results may also differ in settings with a higher prevalence of STIs, where greater diagnosis rates could lead to more people being prescribed doxyPEP. Further, the impact of doxyPEP on gonorrhea diagnosis would likely be reduced in settings with higher rates of tetracycline-resistant gonorrhea. We did not include cisgender women in our analysis because findings from a recent trial of doxyPEP among cisgender women did not find a reduction in STIs [26]; further work is needed to understand the potential role of doxyPEP for cisgender women. Second, we did not estimate the potential impact of doxyPEP on onward transmission to partners. Given the high rates of STIs within 12 months of a previous diagnosis in our cohort, potentially indicating high rates of reinfection driven by small networks of transmission, we may have underestimated the impact of doxyPEP on population-level STI incidence as interrupting chains of transmission within such networks would likely have population-level benefits. Third, our counterfactual scenarios explored the proportion of people prescribed doxyPEP and the cumulative length of time people were prescribed doxyPEP; we were not able to estimate the number of doxyPEP doses used in each scenario because we did not have data on the number of condomless sex episodes, but participants in clinical trials of doxyPEP reported taking a median of 3.4 to 4.0 doses per month [7, 8]. Last, efficacy estimates were based on clinical trials, where adherence may have differed from clinical practice; our estimates of potential impact may therefore overestimate or underestimate effectiveness during implementation.

## CONCLUSIONS

In this cohort of GBM, transgender women, and nonbinary people who were assigned male sex at birth and accessing STI testing at a US community health center, we estimated that prescribing doxyPEP to individuals with bacterial STIs, particularly concurrent or repeated STIs, could avert a substantial proportion of all STIs. When comparing different strategies for prescribing doxyPEP, the most efficient prescribing strategies were based on STI history rather than HIV status or PrEP use. These findings can inform clinical guidelines for doxyPEP prescribing that balance potential benefits and harms of use. Additional research will be needed to understand and optimize the implementation of doxyPEP, including access, uptake, adherence, impact on STI incidence and antimicrobial resistance, and user-reported outcomes.

## Supplementary Material

ciad488_Supplementary_Data

## Supplementary Data

Supplementary materials are available at *Clinical Infectious Diseases* online. Consisting of data provided by the authors to benefit the reader, the posted materials are not copyedited and are the sole responsibility of the authors, so questions or comments should be addressed to the corresponding author.

## Note

***Financial support*.** M. W. T. was supported by a Fulbright Future Scholarship funded by the Australian-American Fulbright Commission and the Kinghorne Foundation.
