# Supplementary material for: Potential Impact of Doxycycline Post-exposure Prophylaxis Prescribing Strategies on Incidence of Bacterial Sexually Transmitted Infections
Source: Clin Infect Dis. 2023 Aug 18;82(2):304–11. doi: 10.1093/cid/ciad488 (PMC13016692; doi:10.1093/cid/ciad488)
Supplement: ciad488_Supplementary_Data [file ciad488_supplementary_data.docx]

SUPPLEMENTARY MATERIALS

**Potential impact of doxycycline post-exposure prophylaxis prescribing strategies on incidence of bacterial sexually transmitted infections**

Michael W. Traeger, Kenneth H. Mayer, Douglas S. Krakower, Sy Gitin, Samuel M. Jenness, Julia L. Marcus

Table of Contents

[Appendix 1. Syphilis laboratory interpretation algorithm 2](#_Toc141355787)

[Appendix 2. Relative risk reductions used to impute counterfactual diagnosis rates during periods of doxyPEP use 3](#_Toc141355788)

[Appendix 3. Diagnosis rates during the study period by STI, anatomical site, and population group 4](#_Toc141355789)

[Appendix 4. Average number of test events per person per year and time between test events for each subgroup 5](#_Toc141355790)

[Appendix 5. DoxyPEP use, STIs covered, and STI diagnoses averted in counterfactual scenarios in which each doxyPEP prescribing strategy was assumed to have been implemented 6](#_Toc141355791)

[Appendix 6. Proportion of cohort prescribed doxyPEP, proportion of all person-time spent in periods of doxyPEP use, and proportion of all STI diagnoses averted in counterfactual scenarios in which each doxyPEP prescribing strategy was assumed to have been implemented 7](#_Toc141355792)

[Appendix 7. Proportion of cohort prescribed doxyPEP and proportion of STI diagnoses averted in counterfactual scenarios in which each doxyPEP prescribing strategy was assumed to have been implemented, separately for each STI 8](#_Toc141355793)

[Appendix 8. Proportion of STI diagnoses averted (A) and number needed to treat (B) in counterfactual scenarios in which each doxyPEP prescribing strategy was assumed to have been implemented, with each STI-based strategy restricted to PrEP users and/or people with HIV, overall and for each STI (chlamydia, syphilis and gonorrhea) 9](#_Toc141355794)

# Appendix 1. Syphilis laboratory interpretation algorithm

Syphilis algorithm was derived from:

- Menza TW, Berry SA, Dombrowski J, Cachay E, Dionne-Odom J, Christopoulos K, Crane HM, Kitahata MM, Mayer KH. Syphilis Testing and Diagnosis Among People With Human Immunodeficiency Virus (HIV) Engaged in Care at 4 US Clinical Sites, 2014-2018. Clin Infect Dis. 2022 Aug 31;75(3):483-492. doi: 10.1093/cid/ciab944.
- Menza TW, Levine K, Grasso C, et al.  Evaluation of 4 algorithms to identify incident syphilis among HIV-positive men who have sex with men engaged in primary care. *Sex Transm Dis* 2019; 46:e38–41.

**Syphilis Testing**

We defined syphilis testing as any non-treponemal or treponemal test performed on serum within a given 3-month follow-up interval.

**Incident Syphilis**

We defined a case of incident syphilis as having 1 of the 4 following criteria within a given 3-month follow-up interval:

- A rapid plasma reagin (RPR) titer of 1:16 or greater at a patient’s first 3-month follow-up interval during the study period
- A reactive RPR with a titer of 1:4 or greater after a nonreactive RPR in a patient with a history of a reactive RPR during the study period
- A reactive RPR with a titer of 1:1 or greater after a nonreactive RPR in a patient without a history of a reactive RPR during the study period
- A 4-fold or greater increase in RPR titer from 1 follow-up interval to the next follow-up interval

# Appendix 2. Relative risk reductions used to impute counterfactual diagnosis rates during periods of doxyPEP use

|  | HIV-negative PrEP users* | People with HIV^a^ | HIV-negative non-PrEP users^b^ |
| --- | --- | --- | --- |
| Chlamydia | 0.12 | 0.26 | 0.26 |
| Gonorrhea | 0.45 | 0.43 | 0.45 |
| Syphilis | 0.13 | 0.23 | 0.23 |

*a. Risk reduction observed in the US DOXYPEP Study^1^
b. Taken as the lower risk-reduction estimate between HIV-negative PrEP users and people with HIV in the US DOXYPEP study^1^*

Reference for Appendix 2.

1. Luetkemeyer AF, Donnell D, Dombrowski JC, et al. Postexposure Doxycycline to Prevent Bacterial Sexually Transmitted Infections. *N Engl J Med*. Apr 6 2023;388(14):1296-1306. doi:10.1056/NEJMoa2211934

# Appendix 3. Diagnosis rates during the study period by STI, anatomical site, and population group

|  | **Number of people tested at least twice** | **Number of diagnoses** | **Person-years of follow-up** | **Diagnosis rate /100py (95% CI)** |
| --- | --- | --- | --- | --- |
| **Any STI (diagnosis rate)^a^** | 10,546 | 10,144 | 28,275 | 35.9 |
| **Chlamydia** | 9,844 | 4,184 | 26,284 | 15.9 |
| **Rectal** | 7,447 | 3,157 | 19,125 | 16.5 |
| **Urethral** | 9,564 | 1,168 | 25,903 | 4.5 |
| **Pharyngeal** | 3,493 | 106 | 6,372 | 1.7 |
| **Gonorrhea** | 9,941 | 3,652 | 26,466 | 13.8 |
| **Rectal** | 7,447 | 1,887 | 19,120 | 9.9 |
| **Urethral** | 9,603 | 868 | 25,956 | 3.3 |
| **Pharyngeal** | 7,922 | 1,761 | 21,132 | 8.3 |
| **Syphilis** | 9,940 | 1,009 | 27,397 | 3.7 |
| **People with HIV^b,c^** | 1,254 | 1,229 | 3,391 | 36.2 |
| **PrEP users^b,d^** | 5,486 | 5,376 | 13,377 | 40.3 |
| **People without HIV with no PrEP use^b,d^** | 6,147^b^ | 1,026 | 9,508 | 10.8 |

*a. The total number of diagnoses, person-years of follow-up and diagnosis rate for the any STI outcome are not the sum of the respective measures for each STI in the baseline diagnosis rate calculations, as only people with at least 2 STI tests for a given STI are included in STI-specific diagnosis rates. For the diagnosis rate of any STI, and for the main analysis, all people with at least 2 tests events for ANY of the three STI outcomes are included and followed from their first test for any pathogen.
b. Population subgroup disaggregation is for diagnosis of any STI. The total number of people included in the overall cohort is not the sum of the number of people included in each subgroup in the baseline diagnosis rate calculations, as people could be counted in multiple groups over time (e.g., most people entered the cohort not on PrEP and started PrEP during the study period).
c. For baseline diagnosis rate calculations, people were classified as living with HIV from their recorded HIV diagnosis date onwards.
d. For baseline diagnosis rate calculations, people were classified as PrEP users from date of their first PrEP prescription. In the main analysis, prescribing scenarios were restricted to ‘recent PrEP users’ as defined by having a PrEP prescription within 6 months before the indication for the STI-based prescribing strategy.*

*PrEP, preexposure prophylaxis; STI, sexually transmitted infection.*

# Appendix 4. Average number of test events per person per year and time between test events for each subgroup

|  | **Mean number of STI test events per person^a^** | | |
| --- | --- | --- | --- |
| **Year** | **People without HIV and with no PrEP prescription** | **PrEP users** | **People with HIV** |
| **2015** | 1.44 | 2.42 | 1.93 |
| **2016** | 1.48 | 2.62 | 2.09 |
| **2017** | 1.48 | 2.77 | 2.04 |
| **2018** | 1.45 | 2.76 | 2.12 |
| **2019** | 1.44 | 2.89 | 2.17 |
| **2020** | 1.27 | 2.09 | 1.72 |
| **Average across study period^b^** | 1.43 | 2.59 | 2.01 |

*a. Average number of STI test events per person among those with at least one STI test event in the respective year.
b. Mean of the annual mean testing rates per person from 2015-2020*

*Test event was any visit where at least one STI test was performed*

*PrEP, preexposure prophylaxis; STI, sexually transmitted infection*

|  | **Days between STI test events^a^** | | |
| --- | --- | --- | --- |
|  | **People without HIV and with no PrEP prescription** | **PrEP users** | **People with HIV** |
| **Mean** | 245.6 | 120.5 | 168.9 |
| **25^th^ percentile** | 80 | 63 | 68 |
| **Median** | 172 | 93 | 126 |
| **75^th^ percentile** | 338 | 135 | 217 |

*a. Number of days since the previous STI test event for each STI test event. Test event was any visit where at least one STI test was performed.*

*PrEP, preexposure prophylaxis; STI, sexually transmitted infection*

# Appendix 5. DoxyPEP use, STIs covered, and STI diagnoses averted in counterfactual scenarios in which each doxyPEP prescribing strategy was assumed to have been implemented

| **DoxyPEP prescribing strategy** | **Number of people (% of full cohort) prescribed doxyPEP at least once** | **Number of person-years (% of all person-time) of doxyPEP use** | **Number of STIs (% of all STIs) diagnosed during periods doxyPEP use** | **Number of STIs (% of all STIs) averted by doxyPEP** |
| --- | --- | --- | --- | --- |
| (1) Prescribe doxyPEP to all people receiving STI testing | 10546 (100) | 28276 (100) | 10144 (100) | 7182 (70.8) |
| (2) Prescribe doxyPEP to all PWHIV and PrEP users | 6738 (63.9) | 17727 (62.7) | 8322 (82) | 6088 (60) |
| (3) Prescribe doxyPEP to all PrEP users | 5486 (52) | 13357 (47.2) | 6601 (65.1) | 4913 (48.4) |
| Prescribe doxyPEP to **all people** for 12 months following: |  |  |  |  |
| (4) Any STI | 3972 (37.7) | 9091 (32.1) | 5121 (50.5) | 3998 (39.4) |
| (5) Any rectal STI | 2554 (24.2) | 5747 (20.3) | 3951 (38.9) | 2932 (28.9) |
| (6) Gonorrhea diagnosis | 2408 (22.8) | 5079 (18) | 3516 (34.7) | 2598 (25.6) |
| (7) Any 2 STIs past 12 months | 1377 (13.1) | 2914 (10.3) | 2649 (26.1) | 1948 (19.2) |
| (8) Any 2 STIs past 6 months | 1006 (9.5) | 2176 (7.7) | 2193 (21.6) | 1612 (15.9) |
| (9) Syphilis diagnosis | 891 (8.4) | 2077 (7.3) | 1446 (14.3) | 1051 (10.4) |
| (10) 2 concurrent STIs | 733 (7) | 1488 (5.3) | 1554 (15.3) | 1137 (11.2) |
| Prescribe doxyPEP for 12 months to **recent** **PrEP users and people with HIV** following: |  |  |  |  |
| (4) Any STI | 2544 (24.1) | 5389 (19.1) | 3707 (36.5) | 2721 (26.8) |
| (5) Any rectal STI | 1709 (16.2) | 3593 (12.7) | 2915 (28.7) | 2148 (21.2) |
| (6) Gonorrhea diagnosis | 1561 (14.8) | 3151 (11.1) | 2533 (25) | 1863 (18.4) |
| (7) Any 2 STIs past 12 months | 1154 (10.9) | 2402 (8.5) | 2299 (22.7) | 1681 (16.6) |
| (8) Any 2 STIs past 6 months | 775 (7.3) | 1593 (5.6) | 1785 (17.6) | 1301 (12.8) |
| (9) Syphilis diagnosis | 572 (5.4) | 1247 (4.4) | 1011 (10) | 721 (7.1) |
| (10) 2 concurrent STIs | 512 (4.9) | 1021 (3.6) | 1199 (11.8) | 866 (8.5) |
| Prescribe doxyPEP for 12 months to **recent** **PrEP users** **only** following: |  |  |  |  |
| (4) Any STI | 2015 (19.1) | 4116 (14.6) | 2811 (27.7) | 2113 (20.8) |
| (5) Any rectal STI | 1359 (12.9) | 2783 (9.8) | 2173 (21.4) | 1645 (16.2) |
| (6) Gonorrhea diagnosis | 1248 (11.8) | 2444 (8.6) | 1906 (18.8) | 1435 (14.2) |
| (7) Any 2 STIs past 12 months | 930 (8.8) | 1895 (6.7) | 1740 (17.2) | 1304 (12.9) |
| (8) Any 2 STIs past 6 months | 608 (5.8) | 1218 (4.3) | 1317 (13) | 987 (9.7) |
| (9) Syphilis diagnosis | 322 (3.1) | 648 (2.3) | 560 (5.5) | 416 (4.1) |
| (10) 2 concurrent STIs | 367 (3.5) | 705 (2.5) | 822 (8.1) | 613 (6) |

*PrEP, preexposure prophylaxis; STI, sexually transmitted infection.*

# Appendix 6. Proportion of cohort prescribed doxyPEP, proportion of all person-time spent in periods of doxyPEP use, and proportion of all STI diagnoses averted in counterfactual scenarios in which each doxyPEP prescribing strategy was assumed to have been implemented

*PrEP, preexposure prophylaxis; PLWHIV, people living with HIV; STI, sexually transmitted infection.*


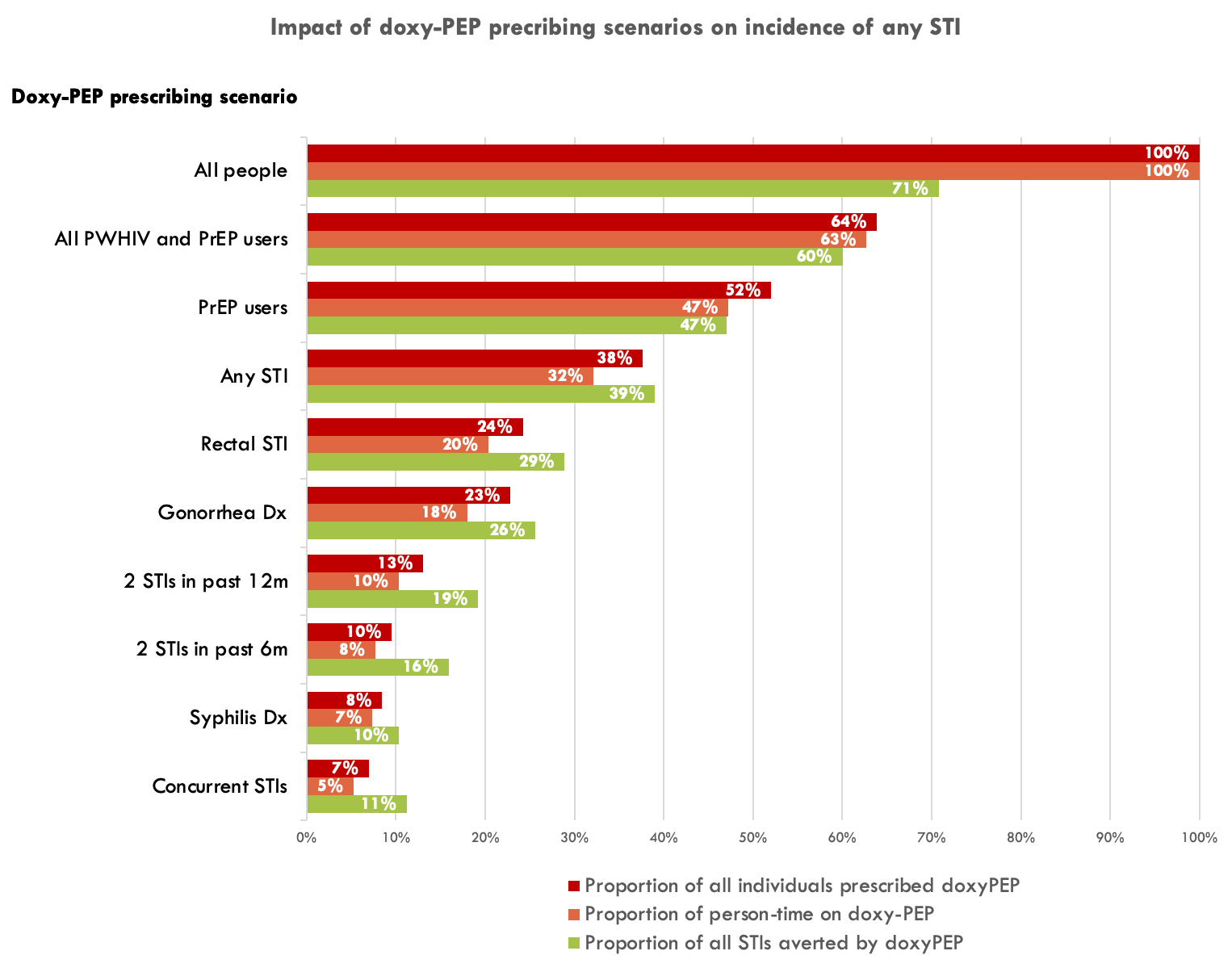


# Appendix 7. Proportion of cohort prescribed doxyPEP and proportion of STI diagnoses averted in counterfactual scenarios in which each doxyPEP prescribing strategy was assumed to have been implemented, separately for each STI

*PrEP, preexposure prophylaxis; PLWHIV, people living with HIV; STI, sexually transmitted infection.*


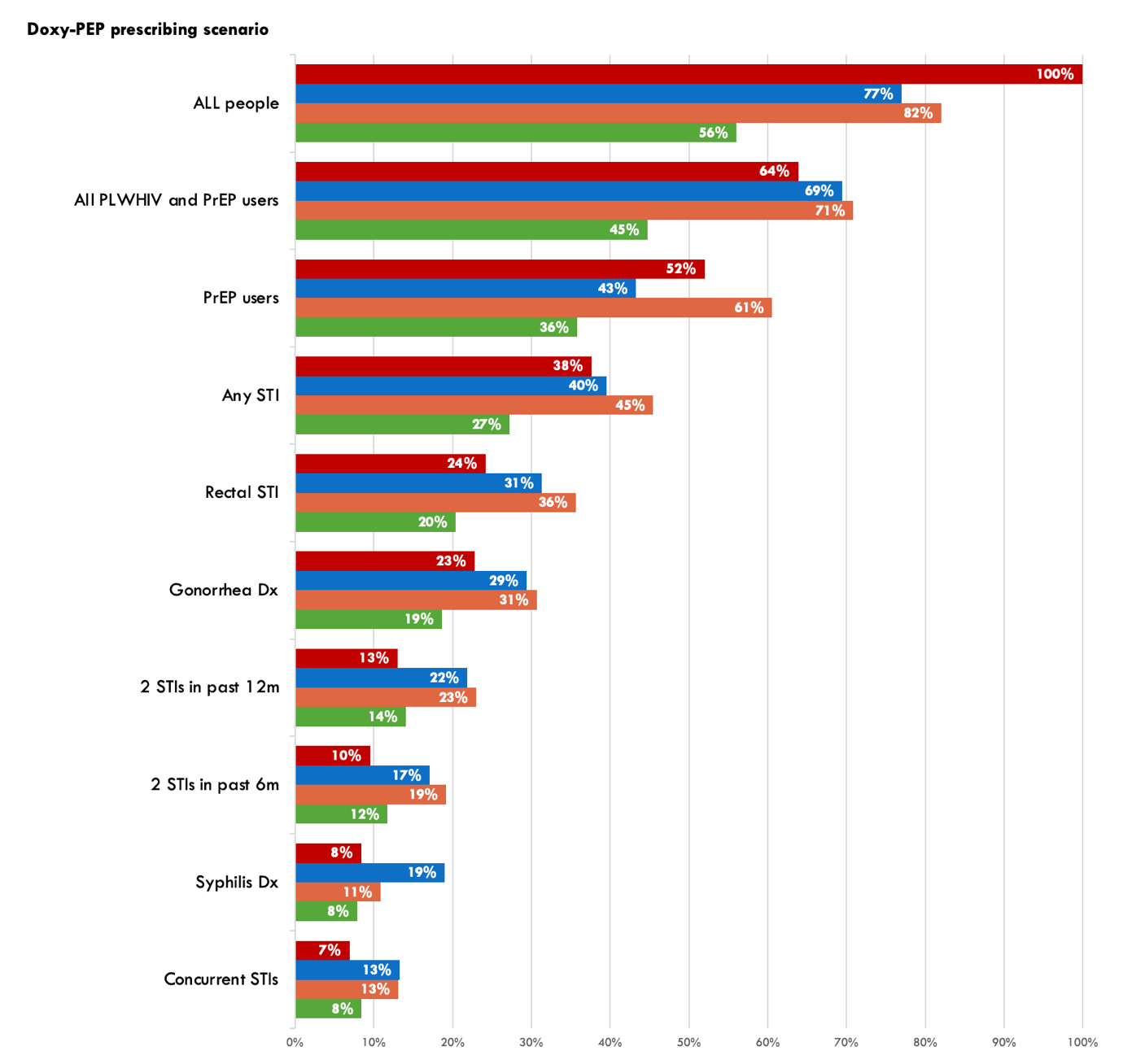


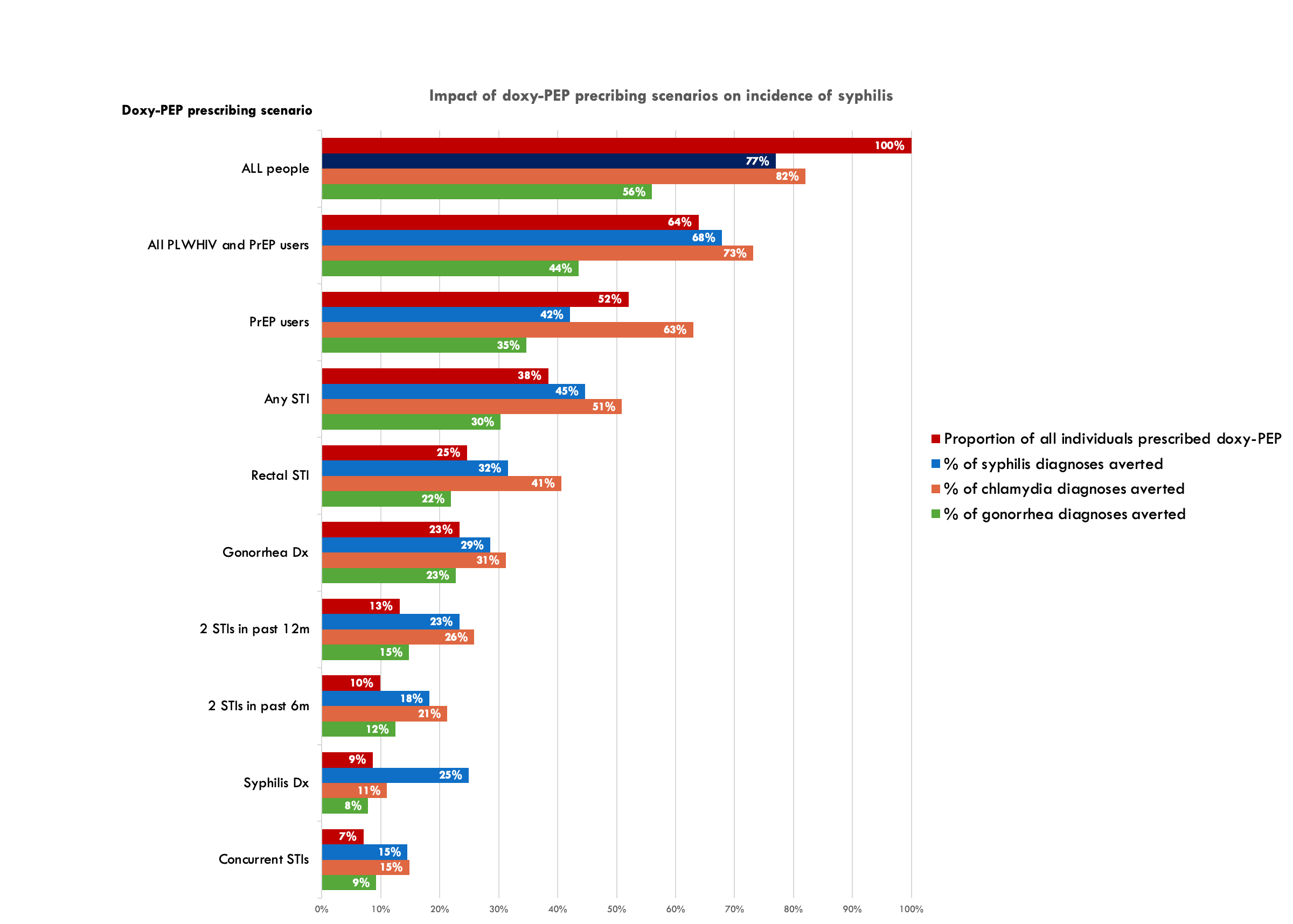


# Appendix 8. Proportion of STI diagnoses averted (A) and number needed to treat (B) in counterfactual scenarios in which each doxyPEP prescribing strategy was assumed to have been implemented, with each STI-based strategy restricted to PrEP users and/or people with HIV, overall and for each STI (chlamydia, syphilis and gonorrhea)

*PrEP, preexposure prophylaxis; PLWHIV, people living with HIV; STI, sexually transmitted infection.*

**Any STI**

**
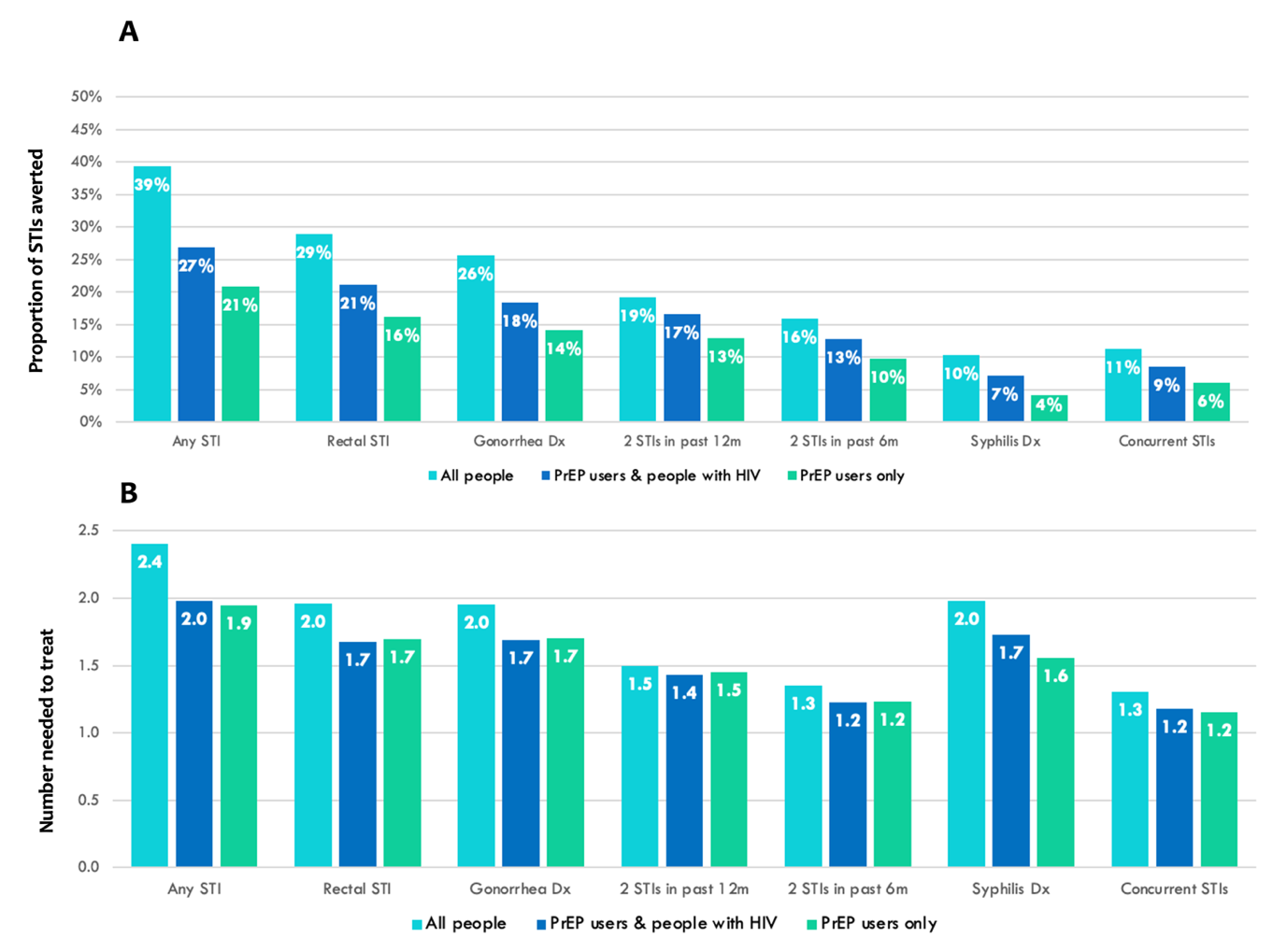
**

**Chlamydia**

**
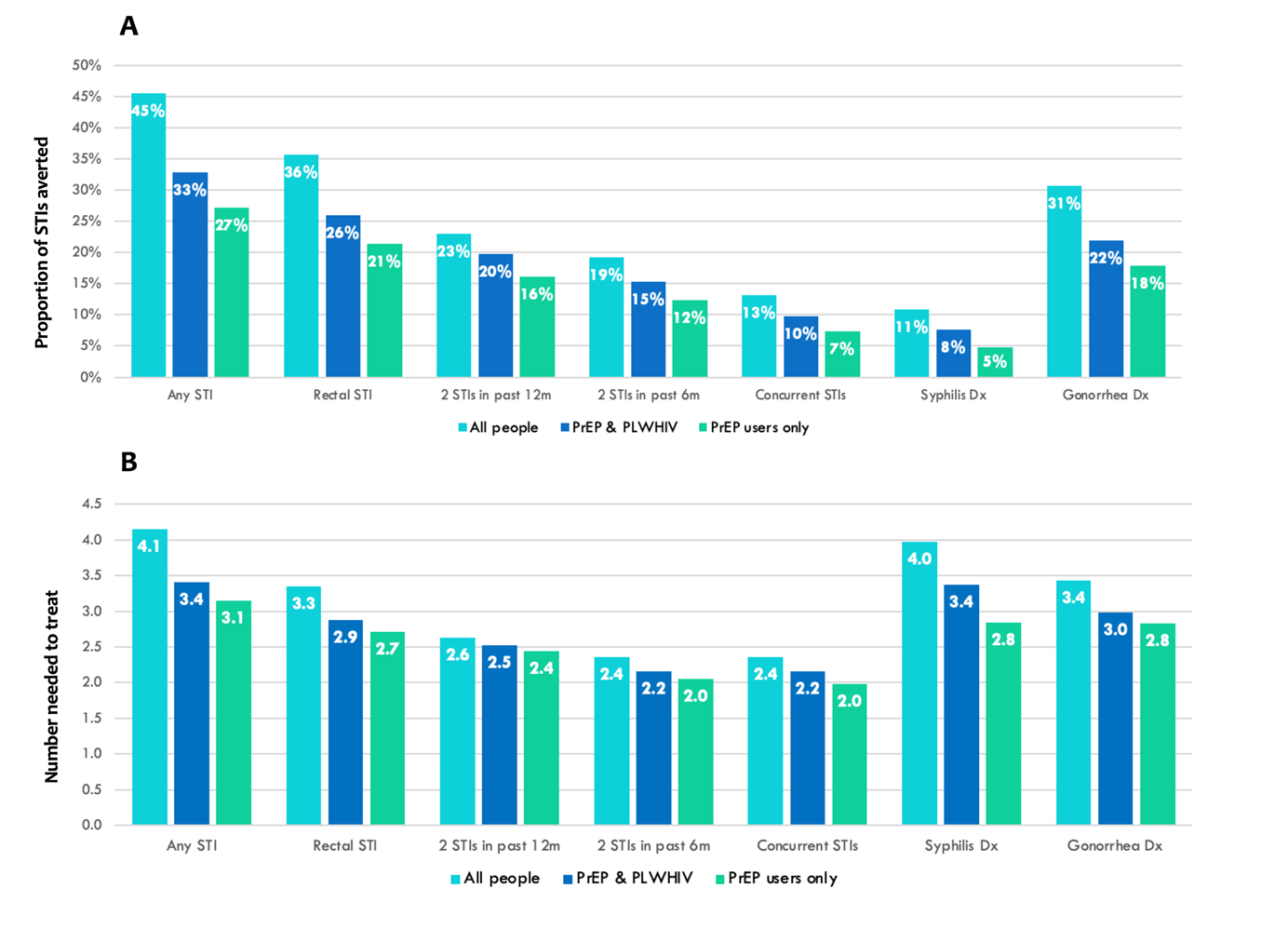
**

**Syphilis**

**
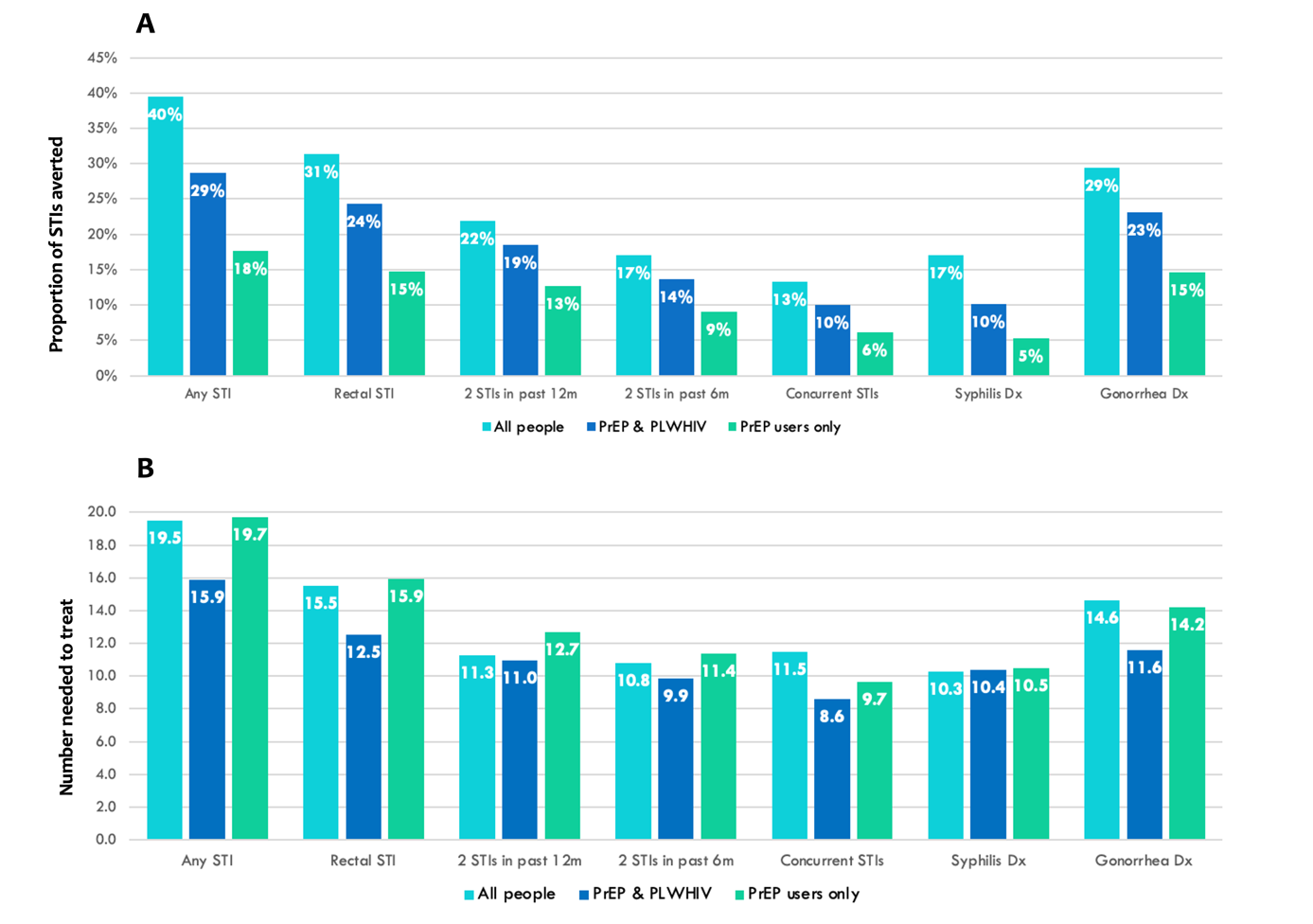
**

**Gonorrhea**

**
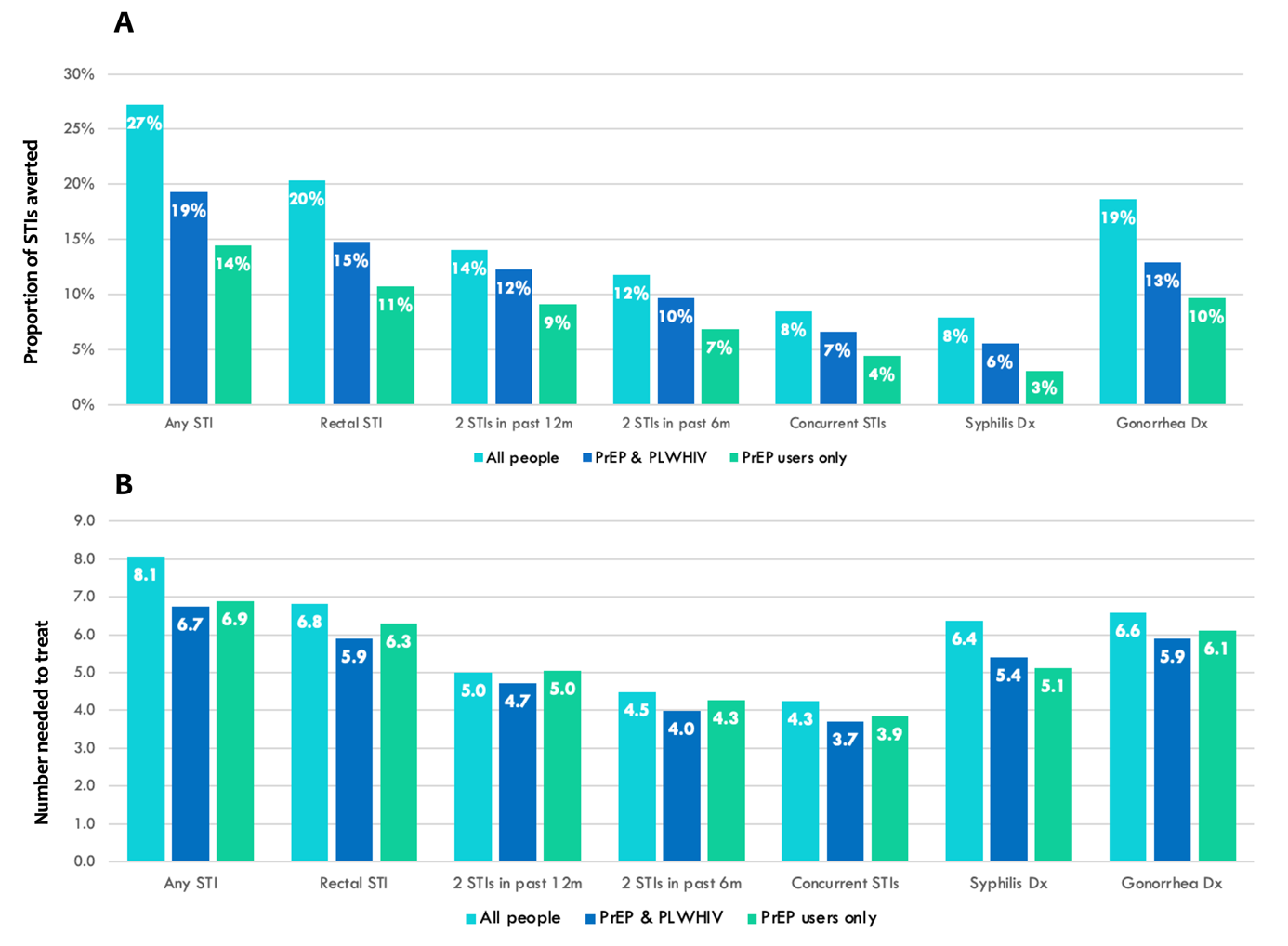
**
